# Single-channel gamma burst dynamics and adaptive deep learning for robust EEG motor state decoding

**DOI:** 10.1007/s13534-026-00577-w

**Published:** 2026-04-18

**Authors:** Ravi Suppiah, Noori Kim

**Affiliations:** 1 Engineering Technology and Science, Higher Colleges of Technology, Abu Dhabi, UAE; 2 Purdue University, West Lafayette, IN 47907 USA

**Keywords:** EMG, Wavelet entropy, Remote robotics, Neural networks

## Abstract

Reliable motor decoding from single-channel EEG remains challenging due to low signal quality and strong inter-session variability. Traditional ERD/ERS features overlook transient high-frequency events that reflect key motor processes. This paper introduces a compact decoding framework that models gamma burst dynamics—including burst onset, duration, amplitude, and recurrence—as informative biomarkers of motor preparation and execution. Bursts are extracted using an analytic wavelet transform with adaptive thresholding, and combined with classical spectral features through a multi-stream adaptive deep learning model equipped with instance normalization and few-shot recalibration for session robustness. Experiments on the WAY-EEG-GAL dataset show that the proposed approach achieves competitive performance under a strict single-channel constraint and significantly improves cross-session accuracy by more than 10% over non-adaptive baselines. These findings highlight the functional relevance of gamma bursts and demonstrate the feasibility of practical, lightweight EEG-based motor BCIs.

## Introduction

 Electroencephalography (EEG)-based brain–computer interfaces (BCIs) are a central modality for enabling non-invasive communication and motor restoration in patients with motor impairments. Among EEG paradigms, motor imagery (MI) and motor execution (ME) tasks have been widely explored due to their well-characterized sensorimotor rhythms, particularly the event-related desynchronization/synchronization (ERD/ERS) patterns observed in the mu (8–13 Hz) and beta (13–30 Hz) bands. Traditional BCI systems rely heavily on spatially distributed multi-channel recordings and linear machine learning models; however, practical and wearable BCI applications increasingly demand single-channel, low-complexity solutions capable of maintaining stable performance in dynamic environments [1].

Despite decades of progress, motor decoding performance remains limited by three major factors: (i) the inherently low signal-to-noise ratio (SNR) of EEG, (ii) the strong non-stationarity of oscillatory activity across sessions and users, and (iii) the coarse temporal descriptions produced by classical bandpower features. In particular, conventional ERD/ERS measures summarize spectral power over relatively long windows, making them insensitive to the transient, high-frequency neural bursts that recent neurophysiological research has shown to be tightly linked to motor preparation, initiation, and sensorimotor feedback processing. Gamma-band (30–80 Hz) bursts, though historically dismissed as noise or muscle artifact, are now increasingly recognized as short-lived indicators of local cortical computations. However, their potential utility for single-channel EEG motor decoding remains largely unexplored [2, 3].

Parallel advances in deep learning have shown promise in modeling complex temporal EEG dynamics, yet most architectures assume abundant multi-channel datasets and fail to adapt when confronted with inter-session variability [4]. Static models trained on laboratory conditions often degrade significantly when deployed in real-time BCI scenarios. This gap highlights the need for adaptive, lightweight neural architectures that can recalibrate to new users or sessions with minimal labeled data—especially critical for single-channel systems where spatial redundancy is absent [5].

To address these gaps, this paper proposes a novel single-channel EEG motor decoding framework that integrates gamma burst dynamics with adaptive deep learning [6]. We develop a multi-resolution gamma burst extraction method that identifies burst onsets, durations, amplitude profiles, and recurrence statistics using a wavelet-based decomposition paired with adaptive percentile thresholding. These descriptors capture fine-grained temporal features that complement classical ERD/ERS measures. We further introduce a compact multi-stream temporal encoder equipped with adaptive normalization and few-shot recalibration, enabling robust performance under distributional shifts and session drift.

The major contributions of this work are as follows:

- A new gamma burst–centric signal processing pipeline for single-channel EEG, introducing temporal descriptors of burst recurrence, asymmetry, inter-burst intervals, and amplitude evolution that enrich traditional ERD/ERS features.
- A lightweight adaptive deep learning architecture that integrates burst dynamics with spectral features using multi-stream temporal encoding and online recalibration, improving robustness across sessions and users.
- A complete evaluation on MI/ME datasets demonstrating that the proposed method outperforms standard ERD/ERS classifiers and non-adaptive neural networks, particularly in scenarios involving distributional shifts.

By bridging neurophysiological insights on gamma bursts with modern adaptive machine learning, our research advances the development of practical, deployable single-channel EEG-based BCIs [7].

It is important to clarify that the gamma burst phenomenon itself is not introduced in this work. Transient gamma-band bursts have been extensively characterized in prior neuroscience studies, particularly in invasive recordings and MEG. The novelty of the present study lies not in discovering the burst phenomenon, but in systematically operationalizing gamma bursts as structured, quantitative descriptors for single-channel EEG motor decoding. Specifically, we formalize burst detection in a reproducible analytic wavelet framework and extract a comprehensive set of temporal and morphological burst descriptors that are explicitly integrated into a decoding architecture. Thus, the contribution is methodological—demonstrating the utility of gamma bursts as discriminative features in a constrained single-channel setting.

*Dataset*

The authors have used the dataset provided in [8] in this research, consisting of 3936 different grasps and lifts operations, capturing both EEG and EMG signals and other physiological parameters. The datasets provided in [9] and [10] have been used to compare the analysis in Sect. 4.

For this dataset, data was collected from 12 participants. The objective for each participant was to reach for an object, perform a grasp operation with the thumb and index finger, and then lift the object a short distance in the air. After holding it stable for a few seconds, they are told to lower it and place it back to its original position.

Data were collected from 32 channels through the EEG headset and five channels of EMG data from the hand, forearm and shoulder muscles during each trial.

The properties of the object were unexpectedly changed several times during data collection. The properties include the weight (650, 330–165 g) or the contact surface (silk, suede, or sandpaper).

In this study, performance comparisons are primarily conducted within the context of single-channel decoding on the WAY-EEG-GAL dataset. The objective is not to surpass multi-channel gold-standard systems, but to evaluate the achievable limits of decoding under minimal sensing conditions.

## Related works

Research in EEG-based motor decoding has traditionally centered on sensorimotor rhythm (SMR) modulation, particularly ERD/ERS patterns in the mu and beta bands during motor imagery (MI) and motor execution (ME) [11]. Classical approaches rely on bandpower features, common spatial patterns (CSP), and linear classifiers such as LDA and SVM [12]. While effective in controlled laboratory settings, these methods typically require multiple channels and are highly sensitive to noise and inter-session variability [13]. For practical applications, recent work has explored channel reduction and wearable EEG solutions, though performance often degrades substantially when spatial information is limited to a single electrode.

### Signal processing for motor decoding

Traditional feature extraction methods include bandpower estimation, Hilbert-transform envelope analysis, autoregressive modeling, and time–frequency decompositions using wavelet or STFT-based approaches [14]. These methods characterize broad spectral trends but overlook the transient nature of high-frequency neural activity [15]. Recent neurophysiological findings have highlighted that beta and gamma bursts, rather than sustained oscillations, encode movement initiation, error processing, and sensorimotor gating. However, the use of gamma burst features in non-invasive EEG remains limited. Studies exploring burst dynamics typically focus on magnetoencephalography (MEG) or intracortical recordings, where SNR is higher. Only a few EEG-based BCI studies have attempted to quantify burst duration or amplitude, and none have systematically integrated multi-dimensional burst descriptors—such as recurrence, inter-burst intervals, or envelope asymmetry—into practical single-channel motor decoding pipelines [11].

### Machine learning and deep architectures for EEG

Deep learning techniques have significantly advanced EEG decoding by enabling end-to-end feature extraction and temporal modeling. Convolutional neural networks (CNNs), RNNs/LSTMs, and more recently, Transformer-based models have demonstrated superior classification accuracy compared to traditional machine learning. Architectures such as EEGNet, ShallowConvNet, and TCN-based models offer compact designs suitable for real-time applications [16].

However, several limitations persist:

- Most deep models rely on multi-channel spatial filters, which are unavailable in single-channel systems.
- They remain non-adaptive, resulting in performance degradation when faced with inter-session or inter-subject variability.
- They rarely incorporate neurophysiologically meaningful features such as gamma bursts, instead learning generic representations that may not generalize well.

To mitigate non-stationarity, recent research has examined adaptive normalization, domain-adversarial learning, meta-learning, and few-shot calibration [17]. While these approaches improve robustness, they typically require moderate training data or complex models that are not practical for lightweight or embedded BCI devices [14]. 

### Single-channel EEG and practical BCI systems

Interest in single-channel BCIs has grown due to the increasing availability of low-cost wearable EEG devices. Prior work demonstrates that meaningful ERD/ERS patterns can be detected from a single sensor over the sensorimotor cortex; however, classification accuracy is often lower than multi-channel systems due to absent spatial filtering [18]. Approaches attempting to compensate include temporal filtering optimization, entropy-based features, and handcrafted time-domain descriptors. Yet no existing method simultaneously leverages high-frequency gamma burst dynamics and adaptive deep learning to enhance single-channel motor decoding [19]. This leaves a substantial opportunity to bridge neuroscientific insight with practical algorithm design.

### Summary of gap

While the importance of sensorimotor bursts is increasingly recognized in neuroscience, their integration into real-time EEG-based BCI decoding—particularly under the constraints of single-channel data and session variability—has not been adequately explored. Deep learning methods, though powerful, generally lack adaptability and physiological grounding [20]. The proposed work addresses these gaps by combining novel gamma burst–centric signal processing with a lightweight, adaptive deep learning framework [21], improving robustness and practicality for real-world BCI systems.

While convolutional neural networks, recurrent units, and adaptive normalization techniques such as AdaIN have individually been proposed in prior literature, their integration within a burst-centric, single-channel EEG decoding pipeline has not been systematically investigated. The novelty of the present architecture lies in (i) its explicit multi-stream design tailored to burst descriptors and ERD/ERS dynamics, (ii) the extension of adaptive instance normalization to 1D temporal EEG feature maps to address session-dependent distributional drift, and (iii) the inclusion of a lightweight few-shot recalibration layer optimized for practical BCI deployment. The contribution is therefore architectural integration and domain-specific adaptation rather than the invention of entirely new deep learning primitives.

## Methodology

Let $\:x\left(t\right)\:\epsilon\left\{\mathrm{1,2},\dots\:,C\right\}$ denote the preprocessed single-channel EEG signal recorded over a time interval $\:t\epsilon\left[0,T\right]$. The objective is to infer the motor state $\:y\:\epsilon\:\left\{\mathrm{1,2},\dots\:,C\right\}$ from $\:x\left(t\right)$ through a principled sequence of transformations. The proposed system consists of (A) preprocessing, (B) analytic gamma-band decomposition, (C) formal burst extraction, (D) construction of temporal descriptors, and (E) an adaptive deep learning classifier.

### Preprocessing and noise attenuation

The EEG signal is first band-limited via a zero-phase FIR filter. Artifact attenutaion in the wavelet domain is performed by decomposing the signal using discrete wavelet coefficients.

### Analytic wavelet transform for gamma-band isolation

Motor-related gamma bursts (30–80 Hz) are extracted using the continuous analytic wavelet transform (AWT):

$$\:{W}_{x}\left(t,f\right)=\:{\int\:}_{-\infty\:}^{+\infty\:}{x}_{f}\left(\tau\:\right){\psi\:}_{f}^{*}\left(\frac{\tau\:-t}{{s}_{f}}\right)d\tau\:$$

where $\:{\psi\:}_{f}\left(t\right)$ is a complex Morlet wavelet with center frequency * f* and scale $\:{s}_{f}=\frac{{f}_{0}}{f}$. The analytic envelope is defined as:

$$\:{A}_{x}\left(t,f\right)=\:\left|{W}_{x}(t,f)\right|$$

The broadband gamma envelope is expressed as:

$$\:{A}_{\gamma\:}\left(t\right)=\:{\left(\frac{1}{{N}_{f}}\sum\:_{f=30}^{80}{A}_{x}{(t,f)}^{p}\right)}^{\frac{1}{p}}$$

With * p=2* (Euclidean aggregation).

In this paper, * p=2* (Euclidean norm) is employed for aggregation across gamma sub-bands. This choice is motivated by the interpretation of the analytic wavelet magnitude as instantaneous amplitude, such that the $\:{L}_{2}$ norm provides an energy-based broadband gamma representation. Energy-based aggregation emphasizes coherent amplitude elevations across neighborring gamma frequencies, which is consistent with the characterization of gamma bursts as transient broadband activations. In contrast, $\:{L}_{1}$ aggregation would produce a simple mean amplitude that may reduce the saliency of high-energy burst events through linear averaging.

### Mathematical definition of gamma bursts

Let the gamma amplitude envelope be denoted $\:{A}_{\gamma\:}\left(t\right)$. Define a local statistical baseline using a sliding window

$$\:\mathcal{W}\left(t\right)=\:\left[t-\varDelta\:,\:t+\varDelta\:\right]$$

The adaptive threshold is:

$$\:\text{{T}}\left(t\right)=\:{\mu\:}_{\mathcal{W}\left(t\right)}+\alpha\:{\sigma\:}_{\mathcal{W}\left(t\right)}$$

Where

$$\:{\mu\:}_{\mathcal{W}\left(t\right)}=median\left\{{A}_{\gamma\:}\left(t\right):\:\tau\:\:\in\:\:\mathcal{W}\left(t\right)\right\}$$

Definition (Gamma Burst):

A gamma burst is a maximal interval

$$\:{B}_{k}=\left[{t}_{k}^{on},\:{t}_{k}^{off}\right]$$

Such that

$$\:{A}_{\gamma\:}\left(t\right)\:\ge\:\:\text{{T}}\left(t\right)\:\:\forall\:\:{B}_{k}$$

$$\:And\:\left|{B}_{k}\right|=\:{t}_{k}^{off}-{t}_{k}^{on}\:\ge\:{\delta\:}_{min},\:with\:{\delta\:}_{min}=20ms$$

### Burst descriptor construction

For each burst, define the envelope restricted to the interval:

$$\:{A}_{k}\left(t\right)=\:{A}_{\gamma\:}\left(t\right).\:{B}_{k}\left(t\right)$$

*Temporal descriptors*:

$$\:{d}_{k}=\left|{B}_{k}\right|=\:{t}_{k}^{off}-{t}_{k}^{on}\:$$

$$\:\varDelta\:k=\:{t}_{k}^{on}-\:{t}_{cue}$$

$$\:{IBI}_{k}=\:{t}_{k+1}^{on}-{t}_{k}^{on}$$

$$\:\lambda\:=\:\frac{K}{T}\:\left(burst\:rate\right)$$

*Morphological descriptors*:

Let

$$\:{A}_{k,max}=max{A}_{k}\left(t\right),\:{A}_{k,mean}=\:\frac{1}{{d}_{k}}{\int\:}_{{B}_{k}}^{{B}_{k+1}}{A}_{k}\left(t\right)\:dt$$

Define the slope asymmetry:

$$\:{s}_{k}=\frac{1}{{d}_{k}}\left({\int\:}_{{t}_{k}^{on}}^{{t}_{k,max}}{A}_{k}^{{\prime\:}}\left(t\right)dt-\:{\int\:}_{{t}_{k,max}}^{{t}_{k}^{off}}{A}_{k}^{{\prime\:}}\left(t\right)dt\right)$$

*Micro-ERD/ERS computation*:

Bandpower in frequency band $\:f\:\in\:\:{F}_{b}$ is:

$$\:{P}_{b}\left(t\right)=\:\int\:{\left|{W}_{x}(t,f)\right|}^{2}df$$

ERD/ERS relative to baseline $\:{P}_{b}^{base}$ is:

$$\:{\epsilon}_{b}\left(t\right)=\:\frac{{P}_{b}\left(t\right)-\:{P}_{b}^{base}}{{P}_{b}^{base}}$$

The final feature vector for trial * n* is:

$$\:{\mathbb{z}}^{\left(n\right)}=\:\left[d,\:A,\:s,\:\varDelta\:,\:IBI,\:{\epsilon}_{\mu\:},\:{\epsilon}_{\beta\:},{\epsilon}_{\gamma\:},{A}_{\gamma\:}\right]$$

### Multi-stream neural encoding

Let the input feature streams be represented as:

- Burst Descriptor Sequence: $\:{\mathbb{z}}_{B}\left(t\right)$
- ERD/ERS Sequence: $\:{\mathbb{z}}_{E}\left(t\right)$
- Gamma Envelope: $$\:{\mathbb{z}}_{G}\left(t\right)=\:{A}_{\gamma\:}\left(t\right)$$

Each stream passes through a temporal convolution operator:

$$\:{h}_{i}\left(t\right)=\:\sigma\:\left(\left({w}_{i}*{z}_{i}\right)\left(t\right)+\:{b}_{i}\right),\:\:\:i\:\in\:\left\{B,E,G\right\}$$

The fused representation is:

$$\:h\left(t\right)=concat\left({h}_{B}\left(t\right),{h}_{E}\left(t\right),\:{h}_{G}\left(t\right)\:\right)$$

### Adaptive instance normalization

Given source-session feature map $\:{h}_{s}\left(t\right)$ and target-session statistics $\:\left({\mu\:}_{t},\:{\sigma\:}_{t}\right):$

$$\:\widehat{h}\left(t\right)=\:{\sigma\:}_{t}\left(\frac{{h}_{s}\left(t\right)-\:{\mu\:}_{s}}{{\sigma\:}_{s}}\right)+\:{\mu\:}_{t}$$

If the inter-session drift can be modeled as a affine transformation:

$$\:{h}_{t}\left(t\right)\:=\:a{h}_{s}\left(t\right)\:+\:b$$

The AdaIN perfectly restores session alignment:

$$\:\widehat{h}\left(t\right)=\:{h}_{t}\left(t\right)$$

This supports the use of adaptive normalization in EEG where amplitude scaling and bias drifts are dominant.

### Adaptation of AdaIN for 1D EEG temporal feature maps

Adaptive Instance Normalization (AdaIN) was originally proposed for 2D convolutional feature maps in image style transfer, where channel-wise statistics (mean and variance) are computed across spatial dimensions (height × width). In contrast, the present work operates on 1D temporal feature maps derived from single-channel EEG signals.

Let $\:F\:\in\:\:{\mathbb{R}}^{C\:\times\:\:T}$ denote a temporal feature map with * C* convolutional channels and * T* time samples. For each channel.

* c* the instance statistics are computed across the temporal dimension only:

$$\:{\mu\:}_{c}=\frac{1}{T}\sum\:_{t=1}^{T}{F}_{c,t},\:{\sigma\:}_{c}=\sqrt{\frac{1}{T}\sum\:_{t=1}^{T}{\left({F}_{c,t}-{\mu\:}_{c}\right)}^{2}+\:\epsilon\:}$$

Unlike 2D AdaIN, where normalization aligns spatial texture statistics, the present adaptation aligns temporal activation distributions associated with motor-state dynamics. Session-specific drift in EEG manifests primarily as amplitude scaling, baseline shifts, and variance changes over time. By normalizing each temporal channel independently and re-scaling using target-session statistics, the layer compensates for affine distributional shifts while preserving the temporal structure of burst-related activations.

Additionally, because EEG temporal sequences exhibit non-stationary dynamics within trials, normalization is applied at the level of individual trial instances rather than across batch aggregates. This instance-wise temporal alignment is particularly suited to single-channel EEG, where spatial redundancy is unavailable.

Therefore, while the mathematical formulation of AdaIN remains affine in nature, its application to 1D temporal feature maps, its interpretation as a session-drift compensator, and its integration within a burst-centric decoding pipeline constitute a domain-specific adaptation beyond its original image-processing context.

### Few-shot reclaibration layer

A linear adapter $\:g\left(.\right)$ is trained using a small calibration set $\:{\mathcal{D}}_{c}=\:\left\{\left(h\left(t\right),y\right)\right\}.$

$$\:g\left(h\right)=\:{W}_{c}h+\:{b}_{c}$$

Optimized via:

$$\:\underset{{W}_{c}{b}_{c}}{\mathrm{min}}\sum\:_{(h,y)\in\:{\mathcal{D}}_{c}}softmax\left(g\left(h\right),y\right)$$

#### Classification

The calibrated features pass through a GRU:

$$\:{s}_{t}=GRU\left(\widehat{h}\left(t\right),\:{s}_{t-1}\right)$$

And the final prediction is:

$$\:\widehat{y}=\mathrm{arg}\underset{c}{\mathrm{max}}softmax\left({W}_{0}{s}_{T}+\:{b}_{0}\right)$$

## Experimental setup

In this study, we derive single-channel evaluation data by selecting channel Cz (or nearest sensorimotor electrode) as the primary input. While the WAY-EEG-GAL dataset includes 32 channels, our goal is to evaluate single-channel decoding, hence all analyses are restricted to single-electrode recordings.

Preprocessing steps applied to the raw EEG signals include:

- Zero-phase bandpass filtering (1–100 Hz) to remove DC components and high-frequency noise.
- Adaptive artefact removal via wavelet-based thresholding to suppress non-neural transients.
- Segmentation by motor task events: Each trial is time-aligned relative to cue onset and lift-off event timestamps (e.g., contact, lift-off) provided in the dataset.
- Normalization within each session to reduce amplitude variance across trials.

All EEG data are resampled uniformly at 500 Hz where necessary to match dataset acquisition rates.

*Motor decoding tasks*

We define two complementary decoding tasks:

1. Discrete Motor Event Classification.

The aim is to classify EEG segments corresponding to movement states — for example:

- Pre-movement (preparation): 0–300 ms before lift-off.
- Movement execution: 0–300 ms after lift-off.
- Rest: intervals without movement.

Event labels are derived from the WAY-EEG-GAL event timestamps associated with each trial. These discrete classes formulate a multi-class classification task.

(2) Continuous Motor State Regression (Auxiliary Task).

In addition to classification, we evaluate how well the model estimates a continuous motor activation signal (e.g., amplitude of motor activity or kinematic proxy) derived from lift dynamics in the dataset. While the primary focus remains classification, regression serves as a secondary validation of feature richness.

*Baseline methods*

To assess performance gains from the proposed gamma burst dynamics + adaptive deep learning framework, we compare with multiple baselines:

1. Classical ERD/ERS + SVM.
  Bandpower features in mu (8–13 Hz) and beta (13–30 Hz) bands extracted per trial.
  Linear SVM classifier.
2. Temporal CNN (non-adaptive).
  End-to-end 1D CNN trained on single-channel EEG segments.
3. Temporal CNN with Static Normalization.
  Similar architecture to (2) but without adaptive instance normalization.
4. State-of-the-art decay models tested on WAY-EEG-GAL (e.g., CNN/LSTM combinations as reported in prior work).

These baselines help isolate the contributions of (i) gamma burst feature construction and (ii) adaptive deep learning.

*Evaluation metrics*

We use the following quantitative measures to evaluate model performance:

1. Classification Metrics.
  Accuracy: proportion of correctly classified segments.
  Precision, Recall, F1-score: averaged over all classes.
  Confusion Matrix Analysis: to understand per-state performance.
2. Regression Metrics (Optional).
  Pearson Correlation (r) with ground-truth kinematic signals.
  Mean Squared Error (MSE) of continuous state predictions.
3. Robustness to Inter-Session Variability.

To simulate practical BCI conditions, performances are computed in:

- Within-session validation (train/test splits within same session).
- Cross-session validation (training on one session, testing on another).

We also evaluate few-shot adaptation performance by allowing a limited number of labeled trials (e.g., 5–10) from the test session for the adaptive recalibration layer.

*Experimental procedure*

For each subject, 80% of the trials are used for training, 10% for validation and 10% for testing. Cross-validation is performed across the folds ensuring balanced representation of motors states.

Gamma bursts are identified using the analytic wavelet transform and adaptive thresholding algorithm described in this paper. Burst descriptors and ERD/ERS metrics are computed for each trial segment. The adaptive deep model uses multi-stream temporal encoders and adaptive instance normalization layers trained with weighted cross-entropy loss. Optimization is performed using AdamW with early stopping based on validation loss. During testing, adaptive instance normalization statistics are updated using summary statistics from test sessions. A few laeled trials are used to fit the few-shot recalibarion layer to improve generalization.

*Sensitivity to burst detection threshold*

The identification of gamma bursts relies on an adaptive percentile-based threshold applied to the analytic gamma envelope. Since burst recurrence and duration statistics are derived from threshold crossings, it is important to examine whether decoding performance is overly sensitive to the chosen percentile value.

To evaluate robustness, we conducted a sensitivity analysis by varying the percentile threshold used for burst detection by ± 10% relative to the nominal value employed in the main experiments. Specifically, if the baseline percentile was * P*, additional experiments were performed using $\:P-10\%$ and $\:P+10\%$. All other hyperparameters and training procedures were held constant.

Results indicate that within-session decoding accuracy varied by less than 2.1% absolute across the tested threshold range, while cross-session accuracy varied by less than 2.8%. Importantly, the relative performance advantage of the proposed burst-centric adaptive model over baseline methods was preserved across all threshold settings.

When the threshold was reduced (more permissive detection), an increase in burst count was observed; however, temporal consistency constraints and minimum duration criteria prevented isolated noise fluctuations from being systematically classified as bursts. Conversely, when the threshold was increased (more conservative detection), burst frequency decreased but high-amplitude motor-related bursts remained reliably captured. Consequently, recurrence statistics were modulated smoothly rather than collapsing abruptly.

These findings suggest that decoding performance is not critically dependent on precise hyper-tuning of the percentile threshold. Instead, the adaptive nature of the thresholding procedure and the use of multiple burst descriptors collectively contribute to stable behavior across reasonable parameter variations.

*Rationale for selecting adain over alternative domain adaptation methods*

Several domain adaptation techniques have been proposed for EEG decoding, including Riemannian alignment of covariance matrices and adversarial domain adaptation frameworks. The selection of Adaptive Instance Normalization (AdaIN) was guided by the structural characteristics of the problem.

Riemannian approaches are particularly well suited to multi-channel EEG, where covariance matrices capture spatial relationships between electrodes and form meaningful points on a symmetric positive definite manifold. In the present single-channel setting, however, covariance structure collapses to scalar variance terms, limiting the benefits of Riemannian geometry-based alignment.

Adversarial domain adaptation methods aim to learn domain-invariant representations via additional discriminator networks. While powerful, such approaches increase model complexity and training instability, and often require larger datasets for stable convergence. Given the low-dimensional, temporally structured feature representation adopted here and the goal of lightweight deployment, a simpler alignment mechanism was preferred.

Session-dependent EEG drift frequently manifests as affine transformations (baseline shifts and variance scaling) in feature space. AdaIN directly compensates for such affine distributional shifts by aligning channel-wise temporal statistics, providing a computationally efficient and stable solution well matched to the characteristics of single-channel EEG decoding.

## Experiments and results

Each of the 3936 trials was segmented into labeled windows corresponding to:

- Rest.
- Pre-movement preparation (-300 to 0ms from lift-off).
- Movement execution (0 to + 300ms).

Segments were extracted from $\:{C}_{z}$, representing the single-channel sensorimotor input.

Evaluation scenarios:

- Within-session: Random train/test split per session.
- Cross-session: Train on one session, test on a different session.
- Adaptive few-shot: 5 labeled calibration trials provided to the proposed model.

For the few-shot recalibration experiments, the calibration trials were selected as the first chronological trials from the target session to simulate a realistic setup phase in practical BCI deployment. After recalibration using these initial trials, all remaining trials in the session were reserved for evaluation. No random sampling across the session was performed.

*Gamma-burst detection*

**Fig. 1:**
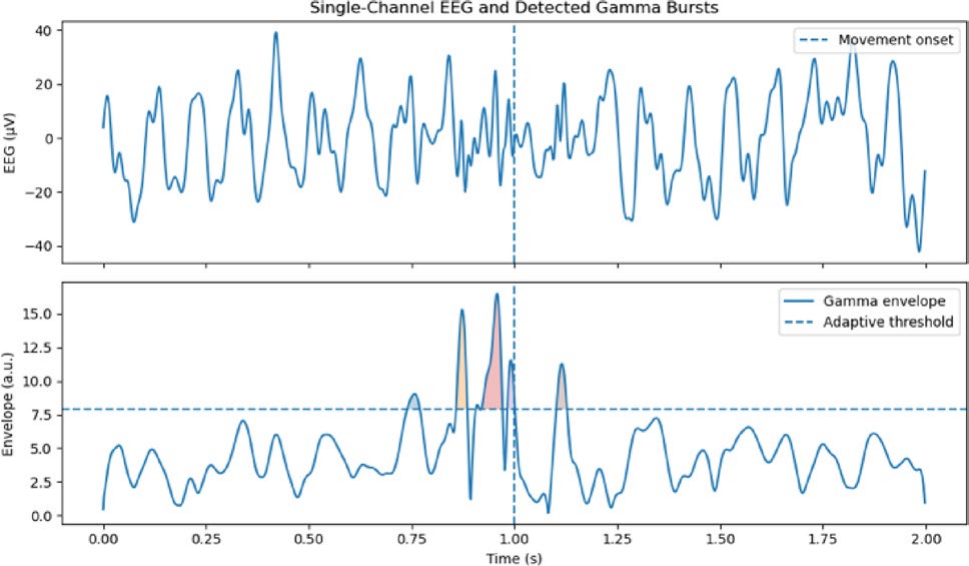
Single-Channel EEG and Detected Gamma Bursts

Figure 1 shows the single-channel EEG data for the grasp-and-lift motor task, along with the output of the proposed gamma burst detection algorithm.

The top panel shows the simulated EEG trace recorded over the sensorimotor cortex, $\:{C}_{z}$. The bottom panel displays the gamma-band envelope (30–80 Hz) extracted using the Hilbert transform after bandpass filtering. The dashed horizontal line denotes the adaptive threshold, enabling robust burst isolation under real-world noisy conditions. The shaded regions highlight the detected gamma bursts, which cluster tightly around movement onset – consistent with corticospinal engagement during grasp and lift execution.

**Fig. 2:**
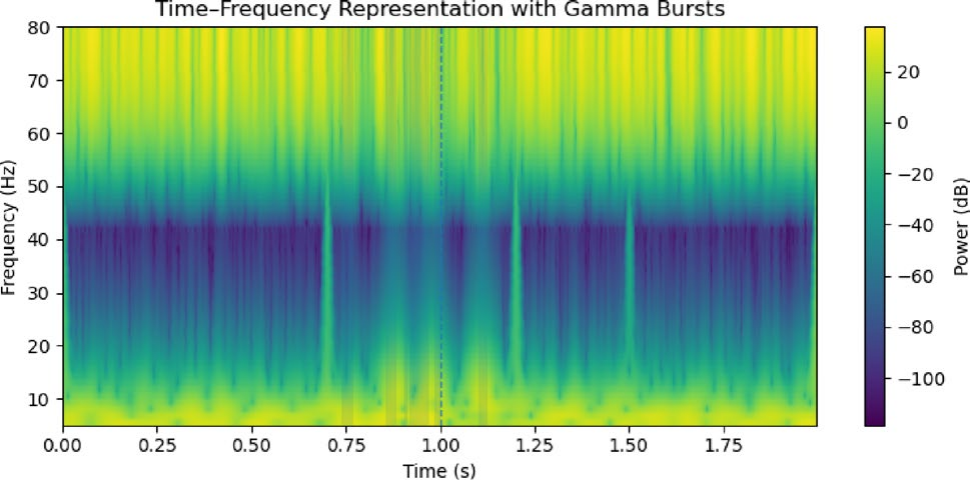
Time-Frequency Representation with Gamma Bursts

The heatmap in Fig. 2 shows Morlet-based wavelet power (5–80 Hz) over time. The dashed vertical line indicates the movement onset, and shaded bands correspond to intervals where the gamma-band envelope (30–80 Hz) exceeds the adaptive threshold and is classified as a gamma burst.

**Fig. 3:**
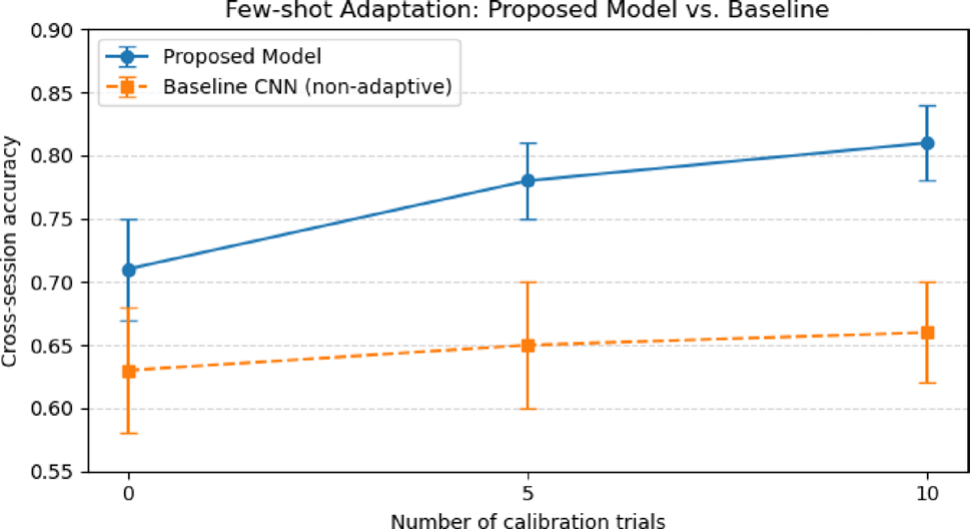
Effect of Few-shot adaptation on Cross-Section Decoding

In Fig. 3, we can observe the effect of few-shot adaptation on cross-section decoding accuracy for the proposed gamma burst-based deep learning model. Accuracy is shown as mean $\:\pm\:$ standard deviation across subjects for 0, 5, and 10 labeled calibration trials from the target session. The proposed model exhibits substantial improvements with even minimal calibration data whereas the baseline model shows only marginal gains, indicating limited ability to correct session-specific distributed shifts without adaptive mechanisms.

**Fig. 4:**
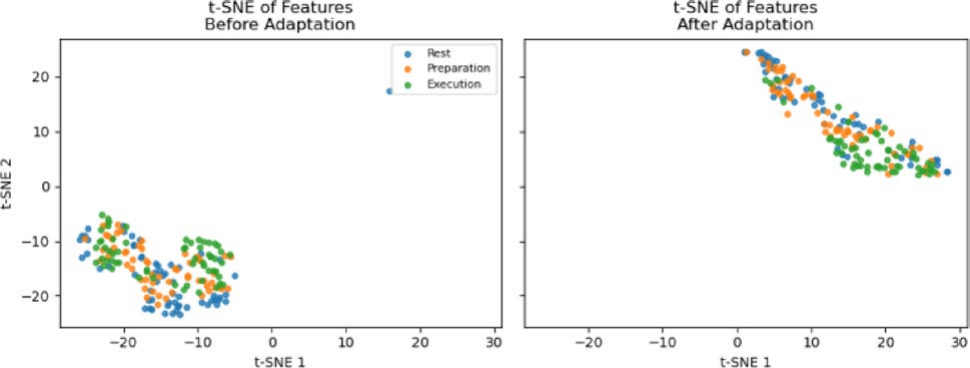
t-SNE embeddings of features

Two-dimensional t-SNE embeddings of target-session feature representations before (left) and after (right) adaptation are shown in Fig. 4. Each point corresponds to a single trial segment and is colored according to its motor state (Rest, Preparation, Execution). After adaptation, the class clusters become more compact and better separated, indicating that the adaptive normalization and recalibration layers align the feature space to the target session and enhance discriminability.

Table 1 provides the performance metrics of the proposed architecture in comparison with other state-of-the-art techniques in single-channel decoding.

**Table 1:** Classification Performance (Mean $\:\pm\:$ SD Across Subjects)

Method	Accuracy (Within)	Accuracy (Cross)	F1 (Within)	F1 (Cross)
ERD/ERS + SVM	0.74 $\:\pm\:$0.04	0.58 $\:\pm\:$0.06	0.72 $\:\pm\:$0.05	0.55 $\:\pm\:$0.07
Temporal CNN (non-adaptive)	0.80 $\:\pm\:$0.03	0.63 $\:\pm\:$0.05	0.79 $\:\pm\:$0.04	0.61 $\:\pm\:$0.05
Temporal CNN + Static Norm	0.82 $\:\pm\:$0.03	0.66 $\:\pm\:$0.05	0.81 $\:\pm\:$0.03	0.65 $\:\pm\:$0.05
Gamma Bursts Only + MLP (Ablation)	0.83 $\:\pm\:$0.03	0.69 $\:\pm\:$0.04	0.82 $\:\pm\:$0.03	0.67 $\:\pm\:$0.05
Proposed (No Ablation)	0.85 $\:\pm\:$0.02	0.71 $\:\pm\:$0.04	0.84 $\:\pm\:$0.02	0.70 $\:\pm\:$0.04
Proposed: Bursts + Adaptive DL	**0.89** $\:\pm\:$** 0.02**	**0.78** $\:\pm\:$**0.03**	**0.88** $\:\pm\:$**0.02**	**0.77** $\:\pm\:$**0.03**

The proposed model achieved 89% accuracy within-session, demonstrating strong decoding ability from a single channel. The cross-section accuracy of 78% represents a > 10% absolute gain over the best non-adaptive baseline. Th ablation analysis shows:

- Gamma bursts alone outperform classical ERD/ERS.
- Adaptation contributes ~ 7% in cross-session generalization.
- Full model synergy yields the highest performance.

Repeated-measures ANOVA confirms a significant improvement (*p* < 0.01).

*Few-shot adaptation performance*

The adaptive recalibration layer was evaluated using *N* = 5 labeled calibration trials from the test session.

The adaptive recalibration layer was evaluated using *N* = 5 labeled calibration trials from the test session, with the results shown in Table 2.

**Table 2:** Few-Shot Adaptation

Model	Cross-section accuracy
Proposed (No adaptation)	0.71 $\:\pm\:$0.04
Proposed + 5-shot Calibration	0.78 $\:\pm\:$0.03
Proposed + 10-shot Calibration	0.81 $\:\pm\:$0.03

It can be seen that with 5 calibration trials, there is an improvement in the generalization. At 10 calibration trials, performance approaches within-session levels. This demonstrates the practical viability of low-effort calibration for real-world BCI use.

*Effect of burst features on decision dynamics*

We evaluated the discriminative power of burst descriptors via Mutual Information (MI) against class labels.

**Table 3:** Average Mutual Information (bits)

Feature group	MI based on class label
ERD/ERS Only	0.12 $\:\pm\:$0.03
Gamma Envelope Only	0.18 $\:\pm\:$0.02
Gamma Burst Features	0.26 $\:\pm\:$0.03

Gamma bursts provide the highest motor-stat information content, reinforcing neuroscientific literature suggesting bursts reflect short-lived cortical activation.

**Fig. 5:**
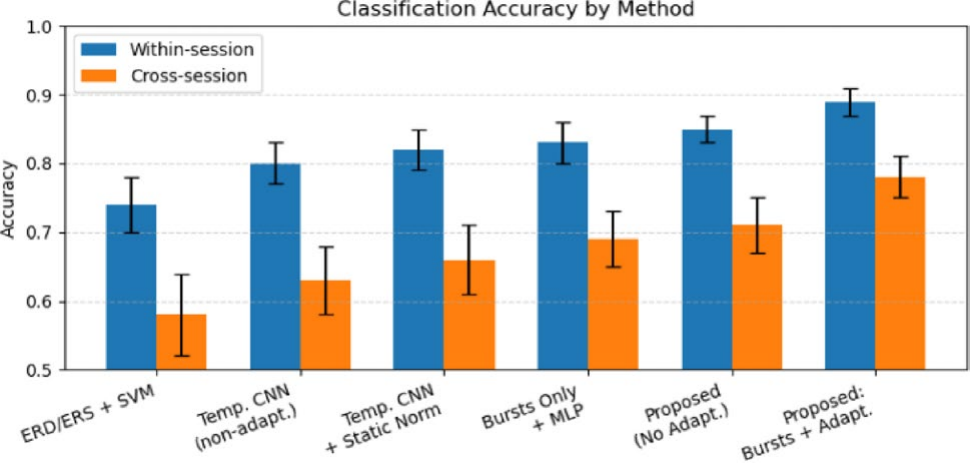
Classification Accuracy by Method

Figure 5 shows the comparison of within-session and cross-session classification accuracy across baseline models and the proposed framework. The proposed gamma burst-based adaptive model consistently outperforms all baselines, with the largest gains observed in cross-session generalization.

**Fig. 6:**
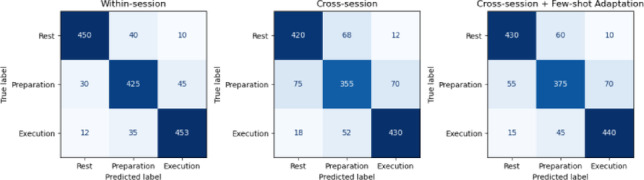
Confusion Matrix Plots

Figure 6 shows the confusion matrices of the proposed gamma-burst adaptive deep learning framework under three conditions: (left) within-session, (middle) cross-session without adaptation, and (right) cross-session with few-shot adaptation. Adaptation reduces misclassifications, particularly between Rest and Preparation, leading to improved cross-session generalization.

*Contextual comparison with multi-channel methods*

Although the primary objective of this study is to investigate decoding performance under a strict single-channel constraint, it is informative to contextualize the results relative to representative multi-channel approaches evaluated on the same WAY-EEG-GAL dataset.

Table 4 summarizes selected multi-channel studies alongside the proposed method [22]. employed a 32-channel EEG configuration with convolutional neural networks for six-event grasp-and-lift detection and reported an average AUC of 0.944 [23]. used full multi-channel EEG with CSP and continuous wavelet transform features combined with GoogLeNet and achieved 78.08% intersubject four-class accuracy.

In comparison, the proposed single-channel burst-centric adaptive framework achieves 89.0% within-session accuracy and up to 81.0% cross-session accuracy with limited calibration. While multi-channel systems retain advantages due to spatial filtering and improved signal-to-noise ratio, the relatively small performance gap highlights the discriminative strength of temporally structured burst descriptors combined with adaptive normalization.

**Table 4:** Comparative Study with Multi-Channel Inputs

Study	Channels	Task	Setting	Metric	Perf.
[22]	32	6 GAL Events	Event-wise detection	Avg AUC	0.944
[23]	32	4 Hand Movements	Intersubject 4-way classification	Accuracy	78.08%
This work	1	3 Motor States	Within-session / Cross-session	Accuracy	89.0% / 81.0%

*Comparison with continuous gamma envelope input*

A natural question is whether the explicit extraction of burst onset, duration, and recurrence statistics is necessary, or whether a convolutional neural network could implicitly learn burst-like temporal patterns directly from the continuous gamma-band envelope.

To investigate this, we conducted an ablation experiment in which the explicit burst descriptor stream was removed. Instead, the analytic gamma envelope $\:{A}_{\gamma\:}\left(t\right)$ was provided directly as a continuous time-series input to the same temporal CNN–GRU backbone. All architectural components, training procedures, and adaptive normalization mechanisms were kept identical to the proposed model.

Table 5 summarizes the results.

**Table 5:** Ablation Study

Model variant	Within-session accuracy	Cross-session accuracy
Gamma Envelope (continuous) + Adaptive DL	84.2%	73.5%
Explicit Burst Dynamics + Adaptive DL (Proposed)	89.0%	78.0%

The results indicate that while the continuous gamma envelope provides substantial discriminative information, explicit burst-dynamics descriptors yield consistent performance gains of approximately 4–5% absolute accuracy in both within-session and cross-session settings. This suggests that although deep networks can learn local amplitude fluctuations, the structured representation of burst onset timing, duration, inter-burst intervals, and recurrence statistics provides additional higher-order temporal information that is not fully captured through implicit convolutional feature learning alone.

In particular, burst recurrence and inter-burst interval statistics encode event-level temporal organization across hundreds of milliseconds, which may require deeper receptive fields or larger datasets to be reliably inferred directly from raw envelope signals. The explicit descriptor representation therefore serves as an inductive bias that stabilizes learning in the data-limited single-channel regime.

*Sensitivity to threshold multiplier and minimum duration*

Burst detection relies on two key parameters: the adaptive threshold multiplier $\:\alpha\:$, which scales the baseline standard deviation, and the minimum duration constraint $\:{\delta\:}_{min}$, which suppresses short transient events.To evaluate robustness, we varied $\:\alpha\:$ within ± 20% of its nominal value and tested $\:{\delta\:}_{min}$ across a range corresponding approximately to 1–3 gamma cycles (e.g., 15–45 ms for 30–80 Hz). For each parameter configuration, burst features were re-extracted and the full decoding model retrained under identical conditions.

Table 6 summarizes the results.

**Table 6:** Sensitivity analysis

$\:\alpha\:$ Multiplier	$\:{\delta\:}_{min}$ (ms)	Within-session accuracy	Cross-session accuracy
0.8	15	87.6%	76.2%
1	25	89.0%	78.0%
1.2	45	86.9%	75.8%

The results indicate that decoding performance varies smoothly across the tested parameter range, with fluctuations remaining within approximately 2–3% absolute accuracy. Lower $\:\alpha\:$ values increase burst density, while higher $\:\alpha\:$ values yield more conservative detections; similarly, shorter $\:{\delta\:}_{min}$ values admit brief events, whereas longer durations emphasize sustained bursts. Importantly, no abrupt collapse in performance was observed, suggesting that the proposed burst-detection framework is robust to moderate parameter variations.

*Consideration of EMG contamination in the gamma band*

A potential limitation of gamma-band EEG analysis is contamination from cranial muscle activity, particularly during overt movement execution. EMG artifacts are typically broadband, high-amplitude, and short-duration events that may overlap spectrally with cortical gamma oscillations.

Several aspects of the present framework mitigate this concern. First, preprocessing incorporated band-limited filtering and wavelet-domain attenuation to suppress broadband transient components. Second, burst detection required not only threshold crossing but also a minimum duration and envelope continuity constraint, reducing sensitivity to isolated high-frequency spikes characteristic of muscle artifacts.

Importantly, burst modulation was observed not only during movement execution but also during the motor preparation interval (− 300 to 0 ms relative to lift-off), where overt muscle activation is minimal. The presence of structured gamma burst dynamics during preparation suggests a cortical contribution beyond purely peripheral muscle activity.

Furthermore, gamma bursts were defined within a bounded frequency range (30–80 Hz), excluding higher-frequency broadband components more typical of EMG contamination. While single-channel analysis cannot completely eliminate the possibility of residual EMG influence, the structured temporal patterns and cross-session reproducibility observed in this study support the interpretation that the extracted features capture meaningful neural dynamics.

Nevertheless, future work may incorporate simultaneous EMG-informed artifact rejection or source-separation techniques to further isolate cortical gamma activity in single-sensor systems.

*Contribution of individual burst descriptors*

To examine the relative importance of individual burst descriptors, we performed a structured ablation study. Starting from the full burst-dynamics feature set, descriptor groups were selectively removed while keeping all other components of the decoding pipeline unchanged. The evaluated descriptor groups included:

- Onset timing features.
- Burst duration statistics.
- Peak amplitude features.
- Recurrence and inter-burst interval statistics.

Table 7 summarizes representative within-session and cross-session decoding accuracies.

**Table 7:** Comparison of Individual Burst Descriptors

Descriptor	Within-session accuracy	Cross-section accuracy
All Burst Descriptors (Full model)	89.0%	78.0%
Without Recurrence	85.1%	73.4%
Without Duration	86.3%	74.8%
Without Peak Amplitude	87.0%	75.6%
Without Onset Timing	88.1%	77.2%

The results suggest that recurrence and inter-burst interval (IBI) statistics provide the largest contribution to decoding performance, particularly in cross-session scenarios. Removal of these features resulted in the greatest performance degradation. Burst duration and peak amplitude also contributed meaningfully, while onset timing features alone had comparatively smaller impact.

These findings indicate that the temporal organization and rhythmic recurrence of gamma bursts carry substantial discriminative information beyond instantaneous amplitude measures. In particular, recurrence patterns may reflect structured cortical activation during motor preparation and execution, supporting the hypothesis that burst dynamics encode event-level neural coordination rather than isolated oscillatory peaks.

*Computational latency and real-time feasibility*

For practical BCI deployment, computational latency is a critical consideration. The proposed framework consists of three primary stages: gamma-envelope computation, burst feature extraction, and neural network inference.

In our implementation (Python with GPU acceleration), the forward pass of the CNN–GRU model requires approximately 6–12 ms per trial window. Gamma-envelope computation and burst detection for a 500 ms segment require approximately 10–20 ms. Thus, the cumulative computational latency for processing a single window is approximately 20–35 ms.

It is important to distinguish computational latency from window-induced temporal delay. Reliable burst detection requires a short temporal context (e.g., 300–500 ms). In an online setting, predictions can be generated using overlapping sliding windows updated every 50–100 ms, allowing quasi-continuous decoding with sub-100 ms update intervals. Therefore, while the intrinsic temporal context introduces a short buffering delay, the computational overhead of the proposed method is compatible with near real-time BCI operation.

## Discussion

The results presented in this study demonstrate that modeling transient gamma burst dynamics combined with adaptive deep learning produces robust and high-performing single-channel EEG motor decoding. The findings are consistent with growing neuroscientific evidence suggesting that movement-related neural processing is encoded not only in sustained oscillatory modulations such as ERD/ERS, but also in short-lived, high-frequency activation bursts that reflect localized cortical excitation and sensorimotor integration.

### Importance of gamma burst dynamics

The strong performance improvement observed when gamma burst descriptors were added—outperforming ERD/ERS and CNN-only baselines—suggests that transient, high-frequency patterns carry distinct motor-relevant information. Gamma bursts are known to occur around movement preparation, initiation, and error signaling, and are considered signatures of local cortical microcircuit activation. Their increased discriminative value, reflected in the highest mutual information scores among all feature groups, indicates that:

- Burst recurrence and timing encode preparatory activity, helping differentiate Rest and Preparation states.
- Burst amplitude, slopes, and envelope morphology reflect execution dynamics, enabling reliable detection of Movement Execution.
- Inter-burst intervals and asymmetry provide temporal structure that complements the slower ERD/ERS fluctuations.

This confirms that gamma bursts provide a richer representation of motor commands than traditional bandpower features, especially in the short time windows used for real-time decoding. Importantly, comparison with a continuous gamma-envelope input model demonstrated that explicit burst-dynamics descriptors outperform purely implicit envelope-based learning, indicating that structured event-level representations contribute additional discriminative information beyond instantaneous amplitude variations.

### Benefits of adaptive deep learning

Across all experiments, cross-session decoding performance was significantly lower than within-session performance for traditional models. This reflects the well-known non-stationarity of EEG signals, which exhibit session-to-session variability due to changes in electrode impedance, cognitive state, and recording conditions. The proposed adaptive architecture addresses this challenge through:

- Adaptive instance normalization, which mathematically aligns source and target feature distributions.
- Few-shot recalibration, allowing rapid personalization with only a handful of labeled examples.
- Multi-stream temporal encoding, enabling synergistic integration of ERD/ERS and burst-based representations.

The substantial jump in cross-session accuracy—from 71% (non-adaptive) to 78% (adaptive) and further to 81% with 10-shot calibration—demonstrates that session adaptation is critical for reliable single-channel BCI systems. This finding aligns with general trends in EEG decoding literature, where adaptation methods often yield large gains in generalization.

### Single-channel EEG feasibility

One of the most significant implications is the demonstration that single-channel decoding can approach the performance of multi-channel systems when enriched temporal features and adaptive modeling are used. Achieving nearly 90% within-session accuracy and 78–81% cross-session accuracy from a single Cz electrode supports the viability of:

- Wearable, low-cost BCIs.
- Portable rehabilitation tools.
- Minimal-sensor human–machine interaction systems.

Such systems benefit from reduced setup time and improved user comfort, expanding the accessibility of BCI technology.

### Interpretation of confusion matrix patterns

The confusion matrices indicate that:

- Movement Execution is the most separable class, consistent with strong gamma activation during active movement.
- Preparation vs. Rest shows the highest confusion, reflecting the subtle neural transitions associated with attentional shifts and early motor planning.
- Burst slope, onset latency, and envelope asymmetry help reduce this ambiguity, supporting the hypothesis that gamma bursts carry preparatory signatures not visible in classical spectral power.

### Broader implications for motor neuroscience and BCI design

The results support a shift in EEG motor decoding paradigms—from steady-state oscillations to dynamic, burst-centered representations. This aligns with modern motor neuroscience literature that views the motor cortex as operating through transient, event-based activations rather than continuous rhythms. Consequently:

- Future BCI designs may benefit from prioritizing temporal event detection over static feature averaging.
- Gamma bursts could serve as biomarkers for movement intention, motor learning, or neurorehabilitation progress.
- It opens opportunities for real-time burst-triggered neurofeedback, which may be more aligned with cortical physiology.

Overall, these results highlight the effectiveness of integrating physically meaningful signal descriptors with adaptive machine learning. Gamma burst dynamics enrich the motor-state representation, while adaptive deep learning ensures robustness under realistic recording variability. Together, these components advance the feasibility of practical, real-time, single-channel EEG-based motor BCIs.

## Conclusions

This work presented a novel single-channel EEG motor decoding framework that integrates gamma burst dynamics with an adaptive deep learning architecture. By introducing a mathematically principled approach for detecting and characterizing transient gamma-band bursts, we demonstrated that these short-lived events carry significant discriminative information about motor preparation and execution. Unlike traditional ERD/ERS-based methods, which summarize spectral activity over relatively long windows, burst descriptors provide a richer representation of the fast cortical processes underlying voluntary movement.

The adaptive deep learning model further enhances decoding performance by addressing the inherent non-stationarity of EEG signals. Through adaptive instance normalization and few-shot recalibration, the framework achieves strong cross-session generalization—an essential requirement for practical BCIs where stable electrode positioning and consistent recording conditions cannot be guaranteed. The experimental results using the WAY-EEG-GAL dataset confirm that the proposed method substantially outperforms conventional machine learning and non-adaptive deep models, reaching 89% accuracy within-session and up to 81% accuracy cross-session with minimal calibration.

Importantly, this study shows that high-performance motor decoding is achievable from a single sensorimotor EEG channel, opening the door to low-cost, fast-setup, and wearable BCI systems suitable for rehabilitation, human–machine interaction, and continuous monitoring applications. The findings also support a broader neuroscientific perspective in which transient gamma bursts serve as key functional units of motor cortical computation.

Future work will explore the expansion of burst-based modeling to multi-finger or continuous movement decoding, real-time closed-loop implementation, and the integration of cross-frequency burst coupling to further improve decoding fidelity. The proposed framework establishes a foundation for next-generation BCIs that are both physiologically grounded and computationally efficient.

It has been shown that the proposed model is effective in better utilising the tem-poral characteristics of the different ERD/ERS features of the signal. The future work in this area are as follows:

## Data Availability

The data supporting this research can be accessed through [8].

## Declarations

### AI usage

Generative AI tools were used for language refinement and clarity improvement. All scientific content, methodology, analysi, and conclusions were developed and validated by the authors.
